# Fluorescence Correlation Spectroscopy in Drug Discovery: Study of Alexa532-Endothelin 1 Binding to the Endothelin ET_A_ Receptor to Describe the Pharmacological Profile of Natural Products

**DOI:** 10.1100/2012/524169

**Published:** 2012-05-01

**Authors:** Catherina Caballero-George, Thomas Sorkalla, Daniel Jakobs, Jessica Bolaños, Huzefa Raja, Carol Shearer, Eldredge Bermingham, Hanns Häberlein

**Affiliations:** ^1^Department of Molecular Pharmacology and Pharmacognosy, Drug Discovery Center, Institute for Scientific Research and High Technology Services, P.O. Box 0843-01103, Panama, Panama; ^2^Smithsonian Tropical Research Institute, P.O. Box 0843-03092, Panama, Panama; ^3^Institute of Biochemistry and Molecular Biology, Rheinische Friedrich-Wilhelms-University, Nussallee 11, 53115 Bonn, Germany; ^4^Department of Plant Biology, University of Illinois at Urbana-Champaign, Room 265, Morrill Hall, 505 S. Goodwin Avenue, Urbana, IL 61801, USA

## Abstract

Fluorescence correlation spectroscopy and the newly synthesized Alexa532-ET1 were used to study the dynamics of the endothelin ET_A_ receptor-ligand complex alone and under the influence of a semisynthetic selective antagonist and a fungal extract on living A10 cells. Dose-dependent increase of inositol phosphate production was seen for Alexa532-ET1, and its binding was reduced to 8% by the selective endothelin ET_A_ antagonist BQ-123, confirming the specific binding of Alexa532-ET1 to the endothelin ET_A_ receptor. Two different lateral mobilities of the receptor-ligand complexes within the cell membrane were found allowing the discrimination of different states for this complex. BQ-123 showed a strong binding affinity to the “inactive” receptor state characterized by the slow diffusion time constant. A similar effect was observed for the fungal extract, which completely displaced Alexa532-ET1 from its binding to the “inactive” receptor state. These findings suggest that both BQ-123 and the fungal extract act as inverse agonists.

## 1. Introduction

The ecological and pharmacological impact of marine organisms is a hot topic in drug discovery programs around the globe. The sustainable use of natural products in investigations aiming at finding new active metabolites with new pharmaceutical and biotechnological applications remains a major focal point of research [1]. Microorganisms, for instance, are targeted as a suitable renewable source of bioactive substances, and the exploration of those microbes coming from unusual habitats like the ocean increases the chances of finding novel drugs [2]. 

Even though current marine pharmacology is mainly centered on the development of analgesic, anticancer, antimicrobial, and cytotoxic agents [3, 4], the effect of marine natural products on other important diseases should be targeted and explored.

The search for selective endothelin ET_A_ receptor antagonists is still a top priority in the discovery of drugs to treat cardiovascular disorders. Endothelin-1 (ET-1), a 21-amino-acid residue peptide, is the most potent vasoconstrictor known [5]. Its effects are mediated via two G-protein-coupled receptors (GPCR), namely, ET_A_ and ET_B_, which are coupled to several subfamilies of the heterotrimeric G protein family, mainly G_q_, G_11_, G_s_, and G_i2_ [6]. During the development of cardiovascular disease, the expression and biological activities of ET-1 and its receptors are altered. Even though mainly ET_A_ receptors are located on vascular smooth muscle cells, a few of the ET_B_ receptors may also be present. Thus, the vasoconstrictor effect caused by ET-1 at this level is mediated by activation of both receptors [7]. The opposite effect is mediated by stimulation of the ET_B_ receptor on the endothelial cells by means of NO and prostacyclin [8]. Due to this situation, the net effect of ET-1 depends not only on the balance between ET_A_ and ET_B_ but also on the receptors localization. The available literature suggests that dual ET_A_/ET_B_ receptor antagonism is more effective than selective ET_A_ receptor antagonism in order to fully prevent the deleterious actions of ET-1 in cardiovascular disease [7]. However, the question remains whether the blockade of ET_B_ is desired, since blocking this receptor delays the clearance of ET-1 in the lungs thus keeping high levels of circulating ET-1 [9].

Adverse effects are relatively common for the majority of the known endothelin receptor antagonists in clinical trials and seem to be related to nonspecific vasodilating effects. The most common clinical adverse events reported have been headache, dizziness, nausea, peripheral edema, nasal congestion, upper respiratory tract infection, sinusitis, dyspnea, and chest pain [10]. For a better understanding on how a drug can cause a pharmacological, a secondary, or an adverse effect, it is important to know the dynamics of the receptor system. Antagonist ligands represent a large proportion of therapeutic agents targeting GPCRs; their interactions with the receptors have been widely characterized in terms of receptor binding and signal transduction [11]. However, the corresponding lateral mobility of the different receptor states involved in the process remains poorly documented [12] promoting the need to carry out more studies to unravel the diffusion characteristics of GPCRs.

**Figure 1:**
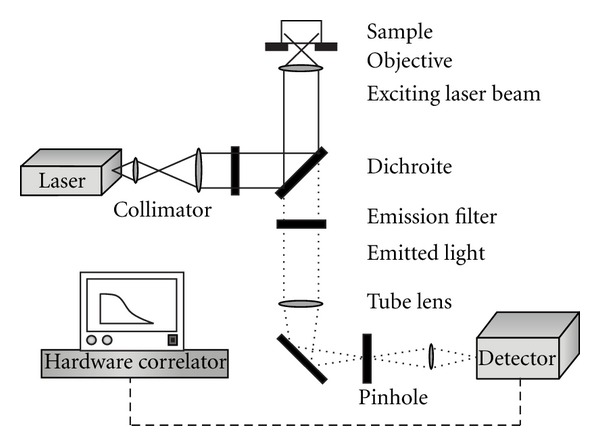
Experimental setup for fluorescence correlation spectroscopy.

Homogeneous receptor assays based on fluorescence like fluorescence correlation spectroscopy (FCS) allow insights into the physiological regulatory mechanisms for membrane receptors at the single molecular level [13]. As it operates in real time and without disturbing the ligand-receptor interaction, FCS is a state-of-the-art tool for drug discovery of natural products and for additional evaluation of their interactions with pharmacological relevant targets on living cells. This method applies statistical analysis for the description of the studied system and for the analysis of the amplitudes of spontaneous fluctuations in the number of particles occurring in a very small volume (fL) of a system to derive conventional diffusion transport and chemical rate coefficients [14]. In measuring ligand-target interactions, autocorrelation of the time-dependent fluorescence signal allows faster diffusing and slower diffusing ligands to be differentiated as to their free and bound state.  Figure 1 shows a typical experimental setup for FCS. FCS has proven to be useful not only in phytopharmaceutical research [15] but also to screen large libraries of molecules with high reproducibility and sensitivity [16].

The present work analyses the binding behaviour of Alexa532-ET1, a newly synthesized fluorescently labelled ET-1 derivative, to the ET_A_ receptor using FCS on living vascular smooth muscle cells. The FCS technique allowed the characterization of the two-dimensional diffusion behavior of different Alexa532-ET1/ET_A_ receptor complexes and of the influence of the ET_A_ selective antagonist BQ123 as well as that of the organic extract of the fungus *Fusicladium *sp. on these receptor-ligand complexes. 

## 2. Materials and Methods

### 2.1. Cell Lines, Chemicals, and Biochemicals

Rat vascular smooth muscle A10 cells were purchased from DSMZ (Braunschweig, Germany); Alexa Fluor 532 carboxylic acid and succinimidyl ester were purchased from Invitrogen-Molecular Probes (Leiden, The Netherlands). BQ-123 and ET-1 were obtained from Sigma (Taufkirchen, Germany).

### 2.2. Biological Material

Fungus *Fusicladium *sp. was isolated from the marine sponge *Amphimedon viridis* collected by SCUBA in Bastimentos Island National Park, Panama, Republic of Panama following the procedure described elsewhere [17]. Colonies on P30 agar (1.25 g/L peptone, 1.25 g/L yeast extract, 3 g/L D-glucose, 20 g/L agar, 30 g/L marine salt) orange brown at first, becoming dark brown in older regions, superficial and immersed, compact with irregular margin, not producing pigments into the agar. Hyphae hyaline, filamentous, septate, branched, containing lipid droplets, not sporulating (See Supplementary Figure  1 in Supplementary material available online at doi:10.1100/2012/524169). After sequencing the ITS1-5.8S-ITS2 gene, the consensus sequence of *Fusicladium *sp. was deposited in GenBank under the accession number JN837045. The ethyl acetate extract of *Fusicladium *sp. was prepared as previously described [17].

### 2.3. Synthesis of Alexa532-ET1

The Alexa532-labeled ET-1 derivative (Alexa532-ET1) was obtained from the reaction of 0.55 mg ET-1 with an excess of activated fluorophore Alexa Fluor 532 carboxylic acid, succinimidyl ester in 225 *μ*L PBS supplemented with 25 *μ*L of 1 M sodium bicarbonate solution to adjust the pH to 8. The ratio of protein/dye was 1 : 2. The mixture was protected from light and stirred at room temperature for one hour. The fluorescently monolabeled ligand was purified by size exclusion chromatography on a Superdex Peptide PC 3.2/30 column, bed dimensions 3.2–300 mm (GE Healthcare Life Sciences, Freiburg, Germany) using PBS as the eluent, at a flow rate of 100 *μ*L/min, and with detection wavelengths of 240 and 525 nm. Identification of monolabeled Alexa532-ET1 was confirmed by MALDI-TOF mass spectrometry on a Voyager STR DE instrument (AB SCIEX, Darmstadt, Germany).

### 2.4. FCS Experiments

FCS measurements were performed via confocal illumination of a volume element of 0.19 fL in a ConfoCor 1 instrument (Zeiss, Jena, Germany) with a C-Apochromat 63x, NA 1.2 objective for water immersion. A dichroic filter and a band-pass filter (FT 540, EF 530–600) (Andover, Salem, MA) separated the excitation light from the emitted fluorescence. Sample excitation was performed with the 514 nm line of an argon laser. The power of the laser beam entering the sample was 2.4 kW/cm^2^. The intensity fluctuations were detected by an avalanche photodiode (SPCM-AQ Series, PerkinElmer Optoelectronics, Fremont, ON) and were correlated with a digital hardware correlator (ALV-5000, ALV, Langen, Germany). To calibrate the volume element of observation for the experiments, a defined concentration of tetramethylrhodamine (TMR) was used. From the determined diffusion time constant of TMR and the diffusion coefficient *D* of 280 *μ*m^2^/s, the radio *ω*_0_ (0.20 *μ*m) and *z*_0_ (1.08 *μ*m) of the volume element were determined. Volume element positioning to the upper membrane of the cell was performed by motor-aided scanning through the cell in the *z*-direction (optoelectronical DC-servodrives, resolution of 0.1 *μ*m). For the FCS experiments, the focus was placed at half maximal of fluorescence intensity at the upper membrane, taking in fast diffusing free ligand and slow diffusing receptor-ligand complexes in the plasma membrane.

### 2.5. Cell Culture and Binding Studies

A10 cells were seeded at a density of 5.0 × 10^4^ on 18 mm poly-D-lysine-coated coverslips. The coverslips were placed in a Nunc 12 well plate, and the cells were cultured for 7 days in Dulbecco's Modified Essential Media supplemented with L-glutamine (2 mM), 1% of a solution containing 10000 I.U./mL penicillin and 10000 *μ*g/mL streptomycin (GIBCO, Invitrogen, Karlsruhe, Germany) and 20% foetal calf serum. The cells were grown in 5% CO_2_ at 37°C until confluence was reached.

Prior to the FCS measurements, cells were washed three times with Locke's solution (5 mM HEPES, 154 mM NaCl, 5.6 mM KCl, 1 mM MgCl_2_, 3.6 mM Na_2_CO_3_, 2.0 mM glucose, 2.3 mM CaCl_2_ (pH 7.4)) at 37°C. For the binding studies, the coverslips were mounted on a carrier and the cells were incubated for 45 min with 300 *μ*L of Locke's solution containing different concentrations of Alexa532-ET1 (5–55 nM).

To determine the nonspecific binding, 1 *μ*M BQ123 was added to cells which had been preincubated with different concentrations of Alexa532-ET1 for 45 min. The incubation with BQ123 lasted 30 min. Similarly, 1 *μ*g/mL of fungal extract was added to cells preexposed to Alexa532-ET1 for 45 min and allowed to act for 30 minutes more at 20°C.

### 2.6. Inositol Phosphate Determination

The inositol phosphate determination assay was performed using the IP-One ELISA Kit for adherent cells (CISBIO, Cedex, France) following the operation instructions. To determine the agonistic capacity of the newly synthesized Alexa532-ET1 ligand, A10 cells were seeded at a density of 10,000 cells per well into a 96-well plate (Nunc, Langenselbold, Germany) four days prior to the experiment. The test substances were diluted in the stimulation buffer to a final concentration ranging from 0.5 to 50 nM. Cells were incubated for 30 minutes before their lysis. The supernatant was transferred into the ELISA plate where the competitive immunoassay took place. After the reaction was stopped, the optical density corresponding to IP accumulation was read at 450 nm with Bio-Rad Microplate Reader (Bio-Rad, Munich, Germany).

### 2.7. Data Evaluation

The autocorrelation function *G*(*τ*) for *j* different diffusing components in a three-dimensional Gaussian volume element is given by the following equation:

(1) $$G\left(\tau \right)=1+\frac{\sum_{j=1}^{n}Q_{j}^{2}N_{j}}{{\left[\sum_{j=1}^{n}Q_{j}N_{j}\right]}^{2}}\frac{1}{1+\tau /\tau_{D_{j}}}\sqrt{\frac{1}{1+{\left(\omega_{0}/z_{0}\right)}^{2}\tau /\tau_{D_{j}}}}$$

with

(2) $$\begin{array}{c} \tau_{D_{j}}=\frac{\omega_{0}^{2}}{4D_{j}}, \end{array}$$

(3) $$\begin{array}{c} Q_{j}=\sigma_{j}\eta_{j}g_{j}, \end{array}$$

where *N*_*j*_ is the average number of molecules of the species *j* in the volume element, *τ*_*D*_*j*__ is the diffusion time constant of the species *j*, *τ* is the correlation time; *ω*_0_ is the radius of the observation volume in the focal plane, *z*_0_ is the radius of the observation volume in the *z*-direction, *D*_*j*_ is the translational diffusion coefficient of the species *j*; *Q*_*j*_ is the quantum yield factor, *σ*_*j*_ is the absorption coefficient, *η*_*j*_ is the fluorescence quantum yield, and *g*_*j*_ is the fluorescence detection efficiency of the species *j*.

### 2.8. Statistical Data Evaluation

All data points from FCS measurements represent mean values and standard deviations of six independent experiments. The statistical significance of results was proven with one factorial analysis of variance (ANOVA). The results were considered to be significant for *P* values ≤0.05.

## 3. Results

The ligand Alexa532-ET1 was obtained by labelling ET-1 with Alexa Fluor 532 carboxylic acid, succinimidyl ester. The structure of Alexa532-ET1 was confirmed by MALDI-TOF mass spectrometry (*m*/*z* 3099, [M-H]^+^).

**Figure 2:**
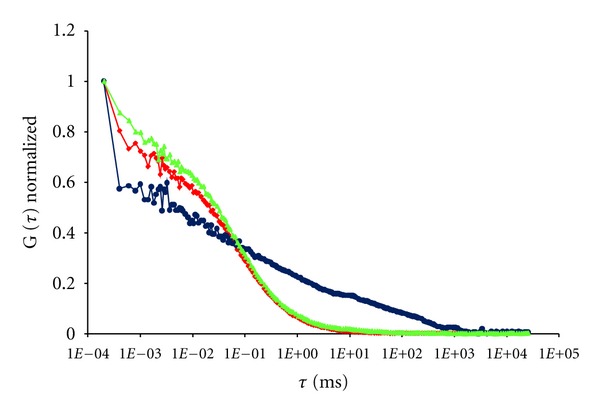
Comparison of autocorrelation curves of the Alexa532-ET1 (14.3 nM) binding to the ET_A_ receptor on living A10 cells. Control experiments (blue), coincubation with 1 *μ*M BQ-123 (green) leads to an autocorrelation curve similar to that of the free ligand (red) indicating the displacement of the bound Alexa532-ET1.

**Figure 3:**
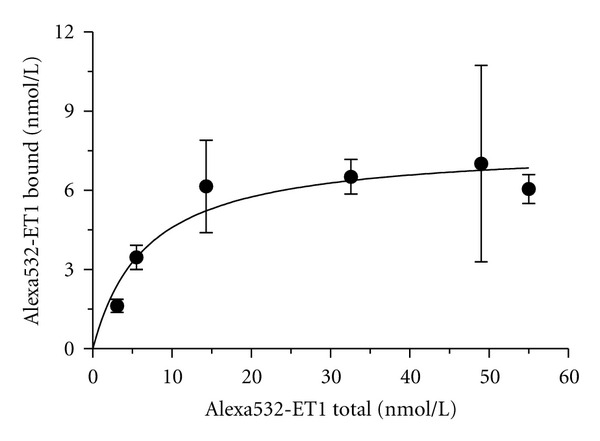
Alexa532-ET1 binding to vascular smooth muscle cells. Averaged bound Alexa532-ET1 concentration versus the total Alexa532-ET1 concentration. The bound Alexa532-ET1 fraction was determined from the autocorrelation function for different Alexa532-ET1 concentrations (*n* = 5).

For the experiment, the illuminated volume element was positioned on the upper plasma membrane of the A10 cell. Forty-five minutes after addition of 14.3 nM Alexa532-ET1 a total binding of 43.5 ± 12.3% (6.22 ± 1.7 nM) was found at 20°C, and two diffusion time constants of *τ*_bound1_ = 63.6 ± 41.6 ms and *τ*_bound2_ = 1.8 ± 0.2 ms (*n* = 6) ms were measured for the lateral mobility of different states of the receptor-ligand complex in the plasma membrane (Figure 2). The diffusion time constant *τ*_free_ of 95.6 ± 9.0 *μ*s (*n* = 6) for the free diffusing Alexa532-ET1 in solution was measured in independent experiments and was kept constant in all fitting procedures. Diffusion coefficients were calculated from the diffusion time constants using (2): *D*_free_ = 104.2 ± 9.0 *μ*m^2^/s for free diffusing Alexa532-ET1, *D*_bound1_ = 0.16 ± 0.06 *μ*m^2^/s for receptor-ligand complexes with hindered lateral mobility, and *D*_bound2_ = 5.6 ± 0.6 *μ*m^2^/s for receptor-ligand complexes with unrestricted mobility. Saturation of Alexa532-ET1 binding was observed at a total concentration of approximately 35 nM. The dissociation constant *K*_*d*_ = 6.77 ± 2.54 nM and the maximum number of binding sites *B*_max⁡_ = 7.70 ± 0.74 nM were obtained from a plot of the bound ligand versus the total amount of the ligand by nonlinear curve fitting (Figure 3). The maximum concentration of bound ligand (*B*_max⁡_) is the same as the maximum number of binding sites in the sample and corresponds to receptor density in the cell membrane. For an average observed membrane area of 0.13 *μ*m^2^ (*πω*_0_^2^), a receptor density (*r*) of 59.2 ± 5.7 receptors/*μ*m^2^ (*n* = 6) (*B*_max⁡_)/(*πω*_0_^2^) was found.

**Figure 4:**
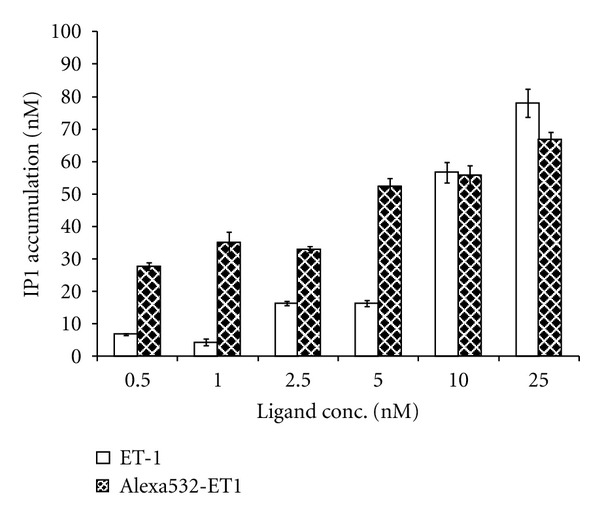
Results of the inositol phosphate (IP1) production assay after stimulation of A10 cells with different concentrations of nonlabeled endothelin 1 and Alexa532-ET1. Data are presented as the mean ± SEM of eight measurements.

In equilibrium, 6.22 ± 1.7 nM of 14.3 nM Alexa532-ET1 was bound to the ET_A_ receptor (Table 1). While 2.3 ± 0.7 nM of the bound ligand (=16.0 ± 5.2% of total Alexa532-ET1) showed hindered diffusion behavior, represented by *D*_bound1_; 3.9 ± 1.0 nM of receptor-ligand complexes (=27.5 ± 7.0% of total Alexa532-ET1) showed unrestricted lateral mobility, represented by *D*_bound2_. Nonlabeled ET-1 as well as Alexa532-ET1 showed a dose-dependent increase of inositol phosphate (IP1) production up to 70–80 nM in A10 cells (Figure 4). In the same line, the binding of 14.3 nM Alexa532-ET1 was inhibited by 1 *μ*M of the selective ET_A_ antagonist BQ-123 (only 82%± or 1.1 ± 0.3 nM of the remaining binding was nonspecific) confirming the specific binding of Alexa532-ET1 to the ET_A_ receptor on A10 cells (Table 1).

After 10 min preincubation with 1 *μ*M BQ-123, the time-dependent binding of 5 nM Alexa532-ET1 was investigated, and after 15 min a total binding of 14.4 ± 5.1% was found. At 30 min a total binding of 32.9 ± 3.9% (1.6 ± 0.2 nM of nonspecific binding) was observed and was stable up to 60 min. Remarkably, the binding of the ligand was found only for the unrestricted diffusing receptor-ligand complex with *τ*_bound2_.

Furthermore, the extract of *Fusicladium *sp. (1 *μ*g/mL) inhibited the binding of Alexa532-ET1 (7.4 nM). Whereas the receptor-ligand complex with *D*_bound1_ was completely displaced, 22 ± 6% (1.6 ± 0.4 nM) of the receptor-ligand complex with *D*_bound2_ was found (Table 1).

## 4. Discussion

FCS provided a more complete description of the pharmacological profile of the drug-system complexes considered. With this technique, it was possible to study the lateral mobility of the receptor-ligand complexes in the plasma membrane and recognize how the downstream responses involved in receptor-ligand interactions are capable of influencing these dynamics. The plasma membrane is characterized by complex dynamic heterogeneous distributions of lipids and proteins, which are believed to have functional implications [18]. Only a complete description of the dynamic organization of the signaling partners by investigating their membrane diffusion behavior will lead to a full understanding of GPCR signal transduction mechanisms [8]. These mechanisms control physiological and side effects of any drug, as illustrated by studies on *μ* opioid receptors [19].

For a better understanding of the endothelin system, a homogeneous ET_A_ receptor assay on living A10 cells using the fluorescently labeled ligand Alexa532-ET1 was established and validated. The FCS model on A10 cells showed a receptor density of 59.2 ± 5.7 receptors/*μ*m^2^ which is comparable with 45 ± 11 ET_A_ receptors/*μ*m^2^ from aortas of New Zealand white rabbits [20]. Furthermore, the Alexa532 moiety did not influence the binding behavior of ET-1, since a high binding affinity for Alexa532-ET1 with a *K*_*D*_ = 6.77 ± 2.54 nM was also found, which corresponds to findings made on cloned bovine ET_A_ receptors (*K*_*D*_ = 20 pM to 1 nM) [21]. Throughout the literature, *K*_*D*_ values as low as a few picomolars and as high as a few nanomolars have been reported for endothelin receptor subtypes [21–23]. It is important to point out that ET-1 binding is never analyzed under true equilibrium conditions, since the ET-1/receptor complexes dissociate slowly and incubation times of more than 20 h are necessary to reach thermodynamic equilibrium, which may lead to artificially high apparent *K*_*D*_ values [21].

A displacement experiment using 1 *μ*M of the selective ET_A_ receptor antagonist BQ123 showed a nonspecific binding of 8 ± 2% which clearly demonstrated the specific interaction of Alexa532-ET1 with the ET_A_ receptor. After 45 min incubation with 14.3 nM of Alexa532-ET1, a detailed evaluation of the bound Alexa532-ET1 showed two diffusion time constants of *τ*_bound1_ = 63.6 ± 41.6 ms and *τ*_bound2_ = 1.8 ± 0.2 ms (*n* = 6) associated with two different receptor-ligand states. A similar situation with a slow and fast diffusing receptor-ligand complex was found for different GPCR systems [13], for instance, the *β*_2_-adrenergic receptor in C6 Glioblastoma cells, hippocampal neurons, alveolar epithelial type II cells (A549) [13, 15], and the GABA_A_ receptor in hippocampal neurons [24, 25]. From the diffusion time constants, three different diffusion coefficients (*D*_bound1_, *D*_bound2_, and  *D*_free_) were calculated for the different states of mobility of the receptor-ligand complex and the ligand. The diffusion coefficient found for Alexa532-ET1 (*D*_free_ = 104.2 ± 9.0 *μ*m^2^/s) is comparable to that found for the tetramethylrhodamine derivative (*D*_free_ = 140.5 ± 2.5 *μ*m^2^/s) [22], showing no effect of the selected fluorescent dye on the ET-1 binding characteristics. During the signal transduction, the ET_A_ receptor interacts with several regulatory molecules like phosphatidylinositol-specific phospholipase C, inositol-triphosphate, diacylglycerol [26], cytoskeleton proteins and G-protein-coupled receptor kinases (GRKs). For instance, it has been shown that, in HEK 293 cells transfected with the human ET_A_ and ET_B_ receptors, ET-1-induced desensitization corresponded temporally with agonist-induced receptor phosphorylation, and appeared to involve the action primarily of GRK2 rather than other GRKs or protein kinase C [27]. In addition to receptor desensitization, internalization of the receptor-bound ligand is a common method of signal termination, which, for the case of the ET_A_ receptor, is mediated via caveolae, with subsequent degradation of at least a portion of the bound ligand [28]. These interactions are capable of changing the diffusion behavior of the receptor. To our knowledge, this is the first time the ET_A_ receptor-ligand interactions have been studied on living cells in real time.

FCS allowed the discrimination of two different states for the ET_A_ receptor. However, other techniques such as single particle tracking enable the detection of additional receptor states, since it is possible to track the lateral mobility of a single receptor-ligand complex on living cells [12] and obtain a more complete pharmacological profile of the receptor.

As described above, a hindered diffusing receptor is associated with complex molecular interactions, which can be interpreted as receptors going into internalization processes or “inactive” states, whereas unrestricted diffusing receptors can be identified as receptors in “active” states. For the ET_A_ receptor model described here, as well as for the GABA_A_ and the *β*_2_-adrenergic receptor models described elsewhere [15, 24, 25], the proportion of the fast diffusing receptor-ligand complexes was higher than that of slow diffusing receptor-ligand complexes [15, 24, 25]. These findings support the multistate receptor model, where the population of “active” receptor states increase after agonist binding [29]. After preincubation of the cells with 1 *μ*M BQ123 for 10 min, we investigated the binding behavior of 5 nM Alexa532-ET1 time dependently. After 15 min we found a total binding of 14.4 ± 5.1% which increased up to 32.9 ± 3.9% after 30 min and was then stable after 60 min. Surprisingly, the binding of the ligand was selectively found for unrestricted diffusing receptor-ligand complexes, suggesting that BQ-123 showed a strong binding affinity to the “inactive” receptor state characterized by the slow diffusion time constant *τ*_bound2_. A similar effect was observed for the ethyl acetate extract of the fungus *Fusicladium *sp., which completely displaced Alexa532-ET1 from its binding to the “inactive” receptor state. Interestingly, a substance with a higher affinity for the “inactive” receptor state is known as inverse agonist [30]. Inverse agonism is well known for benzodiazepine receptors as well as many other GPCRs [31]. A previous study showed that GABA_A_ receptor binding studies using FCS on hippocampal neurons revealed an increased binding of Alexa532-muscimol mediated by the positive cooperative activity of coincubated benzodiazepines (e.g., midazolam), which was selectively found in GABA_A_ receptor-ligand complexes with hindered lateral mobility [24]. These findings suggest that both BQ-123 and the fungal extract act as inverse agonists of ET_A_ receptors on A10 cells, a property that can easily be detected by FCS.

The major advantage FCS being over other ligand-receptor binding assays such as radio-receptor assays is that the interaction receptor-ligand is not influenced at any level during the evaluation. The data are evaluated in living vascular smooth muscle cells without disturbance of the binding dynamics, allowing us to show the behavior of the ET_A_ receptor in its natural environment. FCS offers a state-of-the-art tool for drug discovery of natural products and the evaluation of their interactions with pharmacological relevant targets.

Whereas FCS provides average measurements, single particle tracking (SPT) acquires the trajectories of single molecules [12]. SPT studies are more informative regarding the molecular events following the binding of GPCR to antagonist and agonist ligands. Forthcoming research will study the diffusion characteristics of the ET_A_ receptor using SPT and Alexa532-ET1.

G-protein-coupled receptors, such as ET_A_, possess complex multimolecular machinery regulating signal transduction pathways responsible for their biological effects. We believe that understanding the functional dynamics of the ET_A_ receptor will enable us to propose specific targets for the development of more selective antihypertensive drugs.

## Supplementary Material

Supplementary Figure 1 shows an axenic culture of *Fusicladium* sp. (M2033-5-P30), which was identified by studying its morphological characteristics and its total genomic DNA.

## Figures and Tables

**Table 1:** FCS binding studies with Alexa532-ET1 on endothelin ET_A_ receptor.

Binding of Alexa532-ET1 to A10 cells	Free L with *D*_free_ [%]	R-L with *D*_bound1_ [%]	R-L with *D*_bound2_ [%]
Control^(a)^	56.5 ± 8.5	16.0 ± 5.2	27.5 ± 7.0
Displacement with 1 *μ*M BQ-123 after 30 min^(a)^	92 ± 2	5 ± 2	3 ± 1
Displacement with 1 *μ*g/mL fungal extract after 30 min^(b)^	78 ± 6	N.D.	22 ± 6

L indicates Alexa532-ET1 14.3 nM^(a)^, 7.4 nM^(b)^; R-L: the receptor-ligand complex; N.D.: not detectable.
