# Supplementary material for: Fluorescence Correlation Spectroscopy in Drug Discovery: Study of Alexa532-Endothelin 1 Binding to the Endothelin ETA Receptor to Describe the Pharmacological Profile of Natural Products
Source: ScientificWorldJournal. 2012 May 1;2012:524169. doi: 10.1100/2012/524169 (PMC3353486; doi:10.1100/2012/524169)

SUPPLEMENTARY FIGURE 1. Identification of *Fusicladium sp*. Morphological characteristics and total genomic DNA were taken from axenic culture of fungus with code number M2033-5-P30.

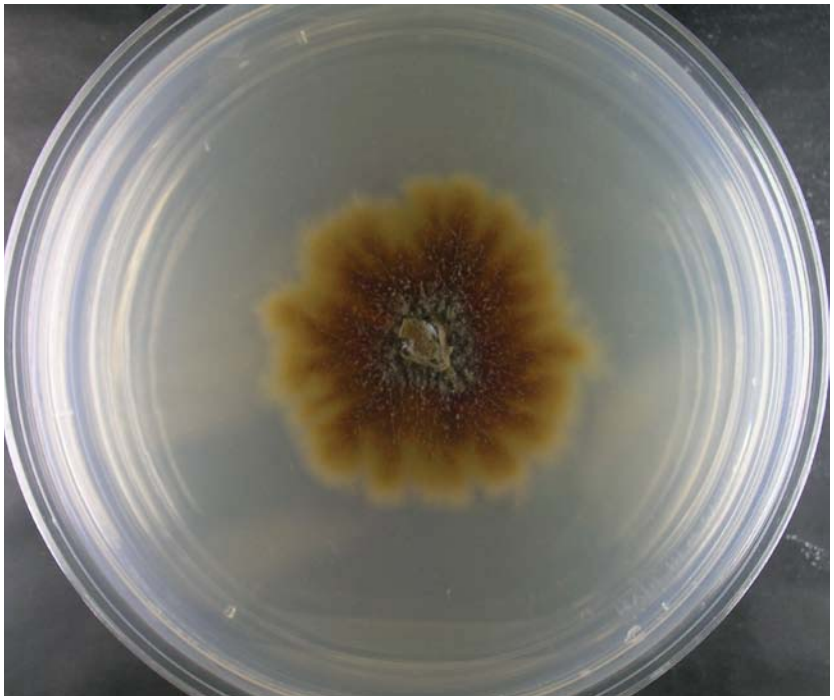

Supplement: Supplementary file 1 — Supplementary Figure 1 shows an axenic culture of Fusicladium sp. (M2033-5-P30), which was identified by studying its morphological characteristics and its total genomic DNA. [file 524169.f1.pdf]
